# A Foundational Study for Normal *F8*-Containing Mouse Models for the miRNA Regulation of Hemophilia A: Identification and Analysis of Mouse miRNAs that Downregulate the Murine *F8* Gene

**DOI:** 10.3390/ijms21165621

**Published:** 2020-08-06

**Authors:** Katarzyna I. Jankowska, Maitreyi Chattopadhyay, Zuben E. Sauna, Chintamani D. Atreya

**Affiliations:** 1 OBRR/DBCD/LCH in the Center for Biologics Evaluation and Research, US Food and Drug Administration, Silver Spring, MD 20993, USA; katarzyna.jankowska@fda.hhs.gov (K.I.J.); maitreyi.chattopadhya@fda.hhs.gov (M.C.); 2 OTAT//DCGT/GTB in the Center for Biologics Evaluation and Research, US Food and Drug Administration, Silver Spring, MD 20993, USA; 3 OTAT/DPPT/HB in the Center for Biologics Evaluation and Research, US Food and Drug Administration, Silver Spring, MD 20993, USA; zuben.sauna@fda.hhs.gov

**Keywords:** microRNAs (miRNAs), bleeding disorders, coagulation factors, hemophilia A, mouse model

## Abstract

Hemophilia A (HA) is associated with defects in the *F8* gene, encoding coagulation factor VIII (FVIII). Our previous studies show that F8-targeting micro RNAs (miRNAs), a group of small RNAs involved in gene regulation, can downregulate F8 expression causing HA in individuals with normal F8-genotypes and increased HA severity in patients with mutations in *F8*. Understanding the mechanistic underpinnings of human genetic diseases caused or modulated by miRNAs require a small animal model, such as a mouse model. Here, we report a foundational study to develop such a model system. We identified the mouse 3′untranslated region (3′UTR) on murine *F8*-mRNA (mu*F8*-mRNA) that can bind to murine miRNAs. We then selected three miRNAs for evaluation: miR-208a, miR-351 and miR-125a. We first demonstrate that these three miRNAs directly target the 3′UTR of mu*F8*-mRNA and reduce the expression of a reporter gene (luciferase) mRNA fused to the mu*F8*-3′ UTR in mammalian cells. Furthermore, in mouse cells that endogenously express the *F8* gene and produce FVIII protein, the ectopic expression of these miRNAs downregulated *F8*-mRNA and FVIII protein. These results provide proof-of-concept and reagents as a foundation for using a normal *F8*-containing mouse as a model for the miRNA regulation of normal *F8* in causing or aggravating the genetic disease HA.

## 1. Introduction

Hemophilia A (HA) is an X-linked bleeding disorder mainly caused by defects in, or a deficiency of, blood coagulation factor VIII. Until recently, it was widely accepted that mutations in the coding sequence of the *F8* gene were the only cause of HA [1]. The mutation types in *F8* include missense, nonsense, synonymous, frameshift, splice site change, the deletion of single or multiple exons and inversions. However, there is no clear correlation between mutation types and HA severity, as the same mutation type (and sometimes the same mutation) is observed in individuals that are diagnosed with mild, moderate or severe HA [2]. More intriguingly, in ~1% of severe and ~3% of mild or moderate HA patients, no mutations in *F8* can be detected, clearly suggesting that there are other molecular mechanisms in addition to the mutations in *F8* that regulate FVIII expression [2,3]. We have previously demonstrated the microRNA-based posttranscriptional downregulation of *F8* [4,5,6] and that this mechanism is observed in HA patients with no defects in the *F8* gene.

Micro RNAs are small, about 22 nucleotides, single-stranded RNAs that have been shown to exert their negative control on protein translation either at the initiation or elongation step by binding mainly to the 3′untranslated region (3′UTR) of their target messenger RNAs [7].

Our previous studies for the first time provided experimental evidence by using HA patient samples that micro RNAs (miRNAs) can downregulate *F8* gene expression and this phenomenon was observed in HA subjects with no mutations in the *F8* gene [5]. 

Available preclinical animal models for HA (rats, dogs, sheep, and genetically engineered mice and pigs) are all centered around developing appropriate therapies for HA [8]. Only recently, we have shown that miRNAs can influence the HA disease outcomes, including in patients with the normal *F8* gene [4,5,6]. To our knowledge, there are no animal models to test this hypothesis towards understanding the mechanistic details of miRNA mediated *F8* dysregulation. As a prerequisite to developing a suitable small animal model such as a mouse, in this report, we first identified the 3′UTR of murine *F8*-mRNA and identified and characterized murine miRNAs that can bind to the mu*F8*-mRNA and dysregulate FVIII expression, so that, based on these foundational studies, a normal *F8*-containing mouse could serve as an animal model for this line of investigations.

## 2. Results

### 2.1. Prediction Analysis and MS2-Tagged Affinity Purification Assays Identify miRNAs Associated with Murine F8 3′UTR

Like in humans, the murine *F8* gene is located on the X chromosome. It spans about 200 kb, and as such is one of the largest genes known. Its transcription yields a 7.5-kb mRNA product. Unlike the 3′UTR of human *F8*-mRNA, that has 1821 nucleotides, the mu*F8*-mRNA has a very short 3′UTR region that is only 125-nucleotides [9]. Nevertheless, like in humans, the mu*F8* gene also comprises of 26 exons, which encode a polypeptide chain of 2319 amino acids (NP_032003) vs. 2351aa in humans (NP_000123)) and exhibit a similar domain structure [9]. We have previously shown that miRNAs are able to target human *F8* 3′UTR and modulate *F8* expression [4,5]. What is interesting is that, in contrast to humans, mice rarely experience spontaneous bleeding episodes [8,9,10,11]; thus, this is an ideal system to evaluate the role of miRNAs in FVIII deficiency and HA.

**Figure 1:**
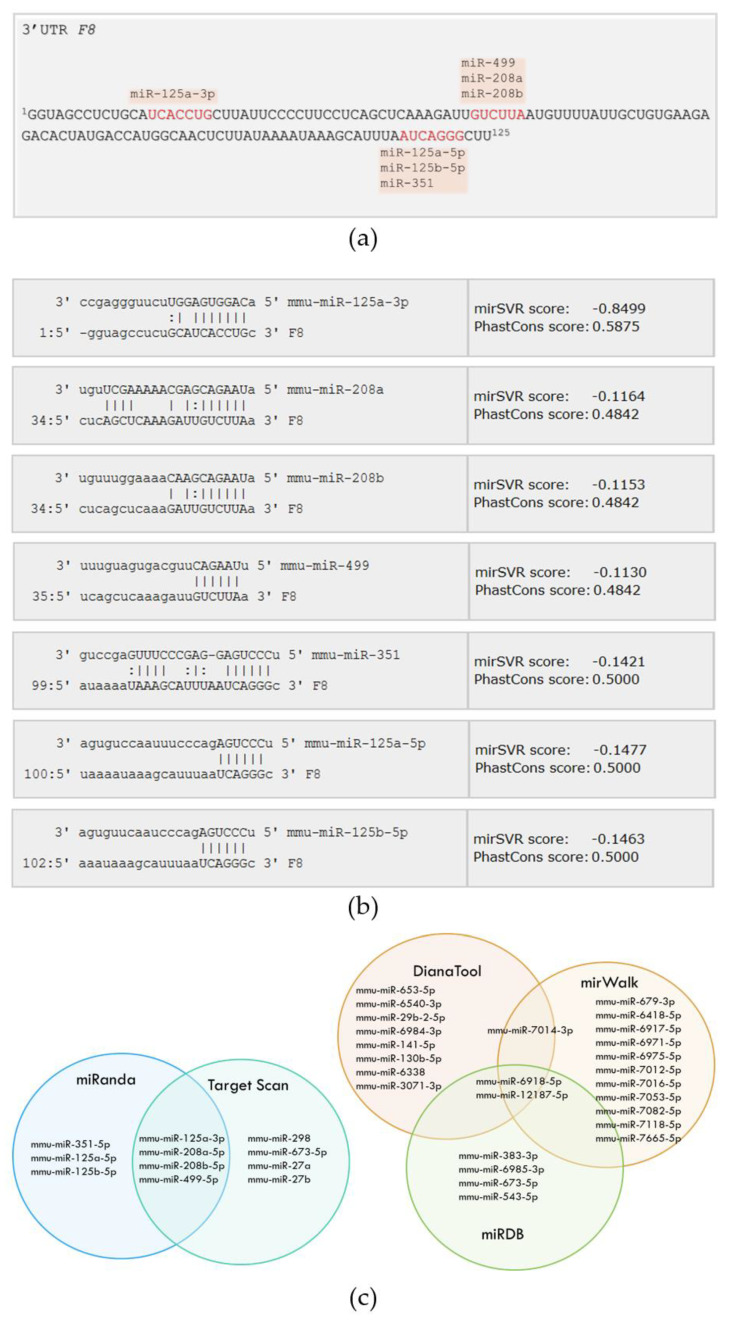
Target gene analysis. (**a**) mRNA sequence of murine 3′untranslated region (3′UTR) of the *F8* region aligned with miRNAs that have potential target sites in *F8* highlighted by red font. (**b**) Sequence alignment of selected miRNAs and mu*F8*-mRNA with potential target sites in the 3′UTR of mu*F8* predicted by miRanda (microrna.org). (**c**) Murine MiRNAs (mmu-miRNA) predicted to target mu*F8* 3′UTR by five different prediction algorithms (miRanda, Target Scan, MiRDB, Diana Tools and mirWalk) and displayed by Venn diagrams.

To identify miRNAs that could potentially bind to the 3′UTR of mu*F8*-mRNA, we used a bioinformatics approach. The software, miRanda (available at microRNA.org) identified six miRNAs that have a binding site in the 3′UTR of the mu*F8* (Figure 1a,b). Multiple software can predict miRNAs, and each have their own advantages and disadvantages; thus, we used multiple computational methods (miRanda, Target Scan, MiRDB, Diana Tools and mirWalk) and observed over 40% overlap between miRNAs identified using miRanda and Target Scan. The three other selected prediction methods (MiRDB, Diana Tools and mirWalk) showed no overlaps with miRanda or Target Scan and only about 20% overlap with each other (Figure 1c).

As our results showing predictive software show, there is considerable diversity in computational predictions, and, thus, miRNAs identified need to be validated experimentally. Thus, an MS2-tagged affinity assay (MS2-TRAP) was performed in HEK293 cells as described previously [12,13,14]. We carried out this experiment by transfecting either MS2 vector alone (MS2-E, negative control) or MS2 fused to the 3′UTR of mu*F8* (MS2-mu*F8*) in HEK293 cells. Using this approach, a pool of endogenous miRNAs associated with the 3′UTR of mu*F8*-mRNA were identified in HEK293 cells. The bound RNAs were then purified and specific miRNAs identified by next-generation sequencing (NGS). The NGS data were analyzed with the miRNA database, miRBase 21. Out of 2588 miRNAs scanned, 375 miRNAs were detected in the MS2 samples. In order to eliminate MS2-bound nonspecific miRNAs in MS2-E samples, we selected miRNAs that were detected with the total number of reads ≥ 40. Out of the 375 miRNAs, 43 miRNAs met this criterion. To further distinguish miRNAs that are significantly different in MS2-E and MS2-mu*F8* samples, we calculated *p*-values using a t-test with degrees of freedom DF = 4 and the assumption that standard deviation equals 8 reads counts. A threshold of *p*-value < 0.05 was used to discriminate between significant and insignificant differences in the detection of miRNA. The data were further sorted by miRNAs fold change. Nine miRNAs displayed a 1.5-fold increases in MS2-mu*F8* (Table 1).

### 2.2. Predicted miRNAs Indeed Target Murine 3′UTR of F8-mRNA

It is important to demonstrate that the miRNAs predicted to target mu*F8* 3′UTR and pulled down in MS2-tagged affinity assay can indeed control the expression of FVIII protein. We selected three miRNAs, miR-208, miR-351 and miR-125a, that were identified in the pull-down assay as well as by in silico prediction, for detailed functional characterization.

**Figure 2:**
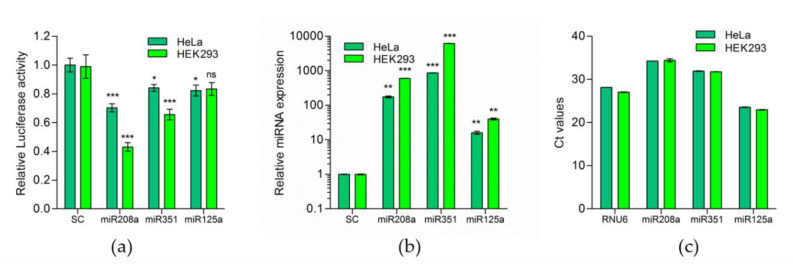
Predicted murine miRNAs indeed target 3′UTR of murine *F8*-mRNA. (**a**) Relative luciferase activity in HeLa and HEK293 cells transfected with luciferase and miRNA expression vectors for miR-208a, miR-351 and miR-125a (HeLa: SC = 1.00 ± 0.049, N = 9; miR-208a = 0.7027 ± 0.02843, N = 9; miR-351 = 0.8413 ± 0.02369, N = 9; miR-125a = 0.8222 ± 0.03816, N = 6; HEK293: SC = 0.9881 ± 0.08065 N = 8. miR-208a = 0.4297 ± 0.02946, N = 11; miR-351 = 0.6558 ± 0.03693, N = 11; miR-125a = 0.8339 ± 0.04463, N = 5.). (**b**) Quantitative polymerase chain reaction (qPCR) analysis to determine the overexpression level of miRNAs 72 h after transfection (Mean ± SEM for HeLa: SC = 1.000 ± 0.01171, N = 3; miR-208a = 174.5 ± 11.15 N = 3; miR-351 = 864.1 ± 9.904 N = 3, miR-125a = 15.98 ± 1.498, N = 3; HEK293: SC = 1.000 ± 0.02263, N = 3; miR-208a = 599.8 ± 1.916, N = 3; miR-351 = 6183 ± 10.04, N = 3; miR-208a = 40.01 ± 2.491 N = 3) and (**c**) Expression (Ct values) of endogenous miRNAs: miR-208a, miR-351 and miR-125 in HeLa and HEK293, relative to RNU6 (HeLa: RNU6 = 28.14 ± 0.015, N = 3; miR-208a = 34.24 ± 0.017, N = 3; miR-351 = 31.92 ± 0.056, N = 3; miR-125a = 23.55 ± 0.052, N = 3; HEK293: RNU6 = 27.06 ± 0.058 N = 3. miR-208a = 34.40 ± 0.37, N = 3; miR-351 = 31.76 ± 0.033, N = 3; miR-125a = 22.95 ± 0.060, N = 3). * *p* < 0.05 ** *p* < 0.01, *** *p* < 0.001; SC = Scrambled miRNA expressing plasmid control.

In two human cell lines, HEK293 and HeLa, we evaluated whether murine miR-208a, miR-351 and miR-125a can regulate the murine *F8* gene (mu*F8*) expression. Cells were co-transfected with luciferase reporter plasmid containing the 3′UTR of mu*F8* with plasmids expressing either miR-208a, miR-351, miR-125a or a scrambled miRNA expressing plasmid control (SC). In both cell lines, decreased luciferase expression (activity) was observed following the co-transfection of miR-208a, miR-351 or miR-125a but not in cells co-transfected with the control scrambled miRNA (Figure 2a). Expression of all three miRNAs, miR-208a, miR-351 or miR-125a, in the transfected cells was confirmed by Quantitative polymerase chain reaction (qPCR) analysis (Figure 2b). The reduction in luciferase activity in HEK293 cells was approximately 60%, 40% and 20% for miR-208a, miR-351 and miR-125a, respectively. The reduction in luciferase activity in HeLa cells was approximately 30%, 20% and 20% for miR-208a, miR-351 and miR-125a, respectively. The changes in luciferase activity were the highest in the presence of miR-208a in HEK293 cells (60% decrease) and the lowest changes were observed for miR-125a (20% decrease). It should be noted that, while all tested miRNAs are endogenously expressed in HeLa and HEK293 cells (Figure 2c), the endogenous levels of miRNA-208a is much lower than that of miR-125a. High expression of miR-125a in both cell lines may be responsible for low and nonsignificant responses in HeLa and HEK293 cells, respectively, as endogenous expression may disturb and interfere with quantification of luciferase signal.

### 2.3. MiR-208, miR-125a and miR-351 Target F8 3′UTR and Downregulate FVIII Expression in Mouse Cells

**Figure 3:**
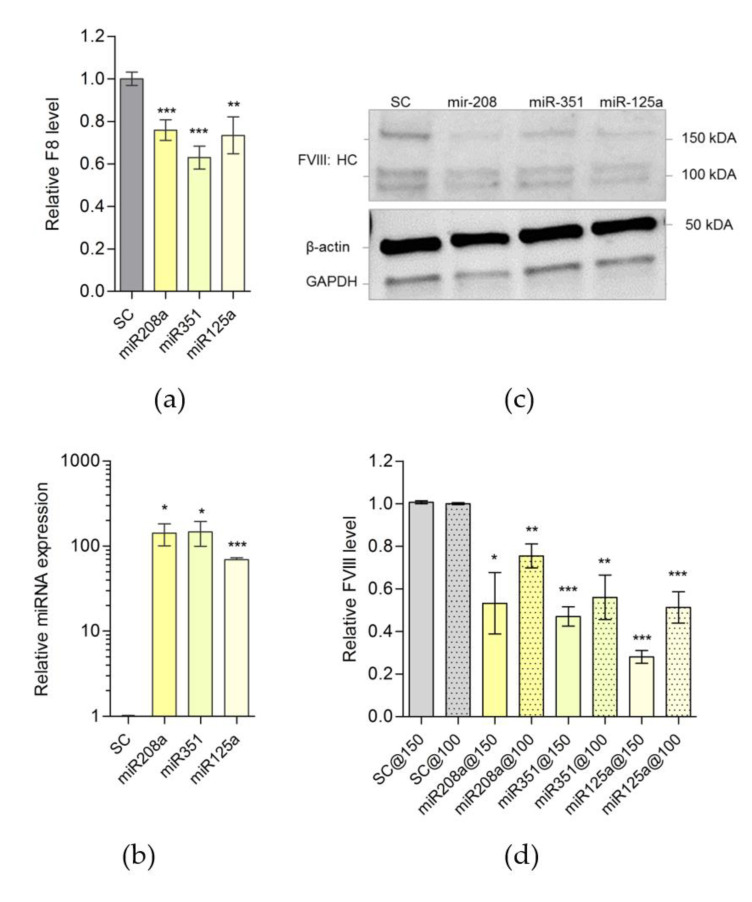
Predicted miRNAs can target *F8* and downregulate FVIII expression in MS1 cells. (**a**) Relative expression of *F8* mRNA (SC = 0.9999 ± 0.03159, N = 19; miR-208a = 0.7589 ± 0.04873, N = 16; miR-351 = 0.6299 ± 0.05398, N = 16; miR-125a = 0.7339 ± 0.08668, N = 16) and (**b**) relative overexpression of miRNAs (SC = 1.001 ± 0.02631 N = 4; miR-208a = 141.5 ± 40.66, N = 4; miR-351 = 146.9 ± 47.05, N = 4; miR-125a = 69.41 ± 3.856, N = 4.) in MS1 cells transfected with miRNAs expression vectors compared to control cells (SC). Q-PCR results of *F8* and miRNAs were normalized to Glyceraldehyde-3-Phosphate Dehydrogenase (GAPDH) and RNU6, respectively. (**c**) Relative FVIII level in MS1 cells collected 72 h after transfection with miRNAs: miR-208, miR-351 and miR-125a expression vectors compared to control cells (SC) determined by Western blot form transfected samples. Multiple species were detected: heavy chain polypeptides (HC) generated after FVIII single chain cleavage within the B domain with an apparent MW range between 90–200 kDa, light chain at about 100 kDa and loading controls: β-actin and GAPDH detected at approximately 42 and 35 kDa, respectively. Full-length blots are presented in Supplementary Figure S1. (**d**) Quantification of FVIII heavy chain in cells transfected with miR-208 and miR-351and 125a with apparent MW of 150 kDa (@150) and 100 kDa (@100); (@100:SC = 1.000 ± 0.004082, N = 4; miR-208a = 0.7545 ± 0.056, N = 4; miR-351 = 0.560 ± 0.104, N = 4; miR-125a = 0.5123 ± 0.07377, N = 4; @150:SC = 1.007 ± 0.006667, N = 3; miR-208a = 0.5320 ± 0.1446, N = 3; miR-351 = 0.4700 ± 0.04583, N = 3; miR-125a = 0.2800 ± 0.0300, N = 3.) from (**c**). Bands normalized to β-actin and GAPDH. * *p* < 0.05, ** *p* < 0.01, *** *p* < 0.001.

Once the effect of downregulation by miR-208a, miR-351 or miR-125a on luciferase expression, when conjugated with 3′UTR of mu*F8* was established, we determined whether the overexpression of tested miRNAs (1) suppresses the *F8*-mRNA levels in the cells or (2) exerts its effect on mu*F8* mRNA translational ability. Mouse MILE SVEN 1 cells (MS1 line) that endogenously express murine FVIII protein [15] were transfected individually with miR-208a, miR-351 or miR-125a expression plasmids and then mu*F8* mRNA levels were estimated by qPCR. As shown in Figure 3a,b, the overexpression of miR-208a, miR-351 and miR-125a significantly (*p* = 0.002) decreased muF8 mRNA levels. Concomitantly, the murine FVIII protein levels, as estimated by Western Blot analysis were also lowered by 25–70% following transfection of miR-208a, miR-351 or miR-125a expression plasmid, respectively, in MS1 cells (Figure 3c,d, Figure S1). These results demonstrate that the expression of miR-208a, miR-351 or miR-125a expression plasmid in mouse cells modulate murine FVIII protein expression.

## 3. Discussion

A growing body of research studies have highlighted the important role of miRNAs in hemostasis and thrombosis [16,17,18,19]. Our previous studies have also demonstrated the dysregulation of some miRNAs in blood samples of HA patients compared to healthy controls and their direct interaction with human *F8*-mRNA [4,5]. These studies were motivated by the unexplained phenomenon wherein some (albeit extremely rare) HA patients with a normal *F8* gene (showing no mutations in the coding and non-coding regions) nonetheless express low amounts of FVIII and exhibit mild, moderate or severe forms of HA. In addition, our studies also provide an explanation for clinical reports showing that HA with the same mutation exhibit different levels of HA severity. We postulate that the miRNA-controlled expression of FVIII may secondarily influence FVIII levels of partially functional FVIII variants, thus aggravating the disease severity.

**Figure 4:**
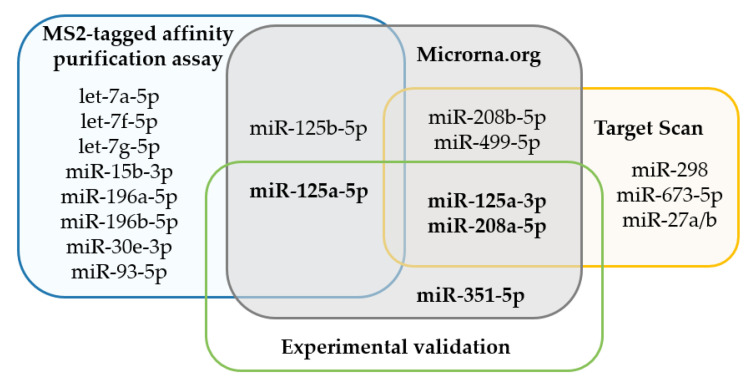
MiRNAs identified to target mu*F8* 3′UTR. Venn diagram of microRNAs identified by MS2-taq affinity assay, predicted to target *F8* 3′UTR by prediction algorithms (microrna.org, http://www.microrna.org), Target Scan and experimentally validated in vitro via luciferase assay and on MS1 cells.

Here, we seek to lay the groundwork for more detailed mechanistic studies of miRNA mediated regulation of FVIII using in vivo mouse models. We therefore used prediction algorithms and an MS2-TRAP assay, to identify and characterize the miRNAs that potentially target and dysregulate murine FVIII (Figure 4). Detailed evaluation of three of these miRNAs were carried out in cell culture systems. We have shown that miR-125, miR-351 and miR-208 directly target the 3′UTR of mu*F8* and downregulate the mu*F8* gene and FVIII in a cell system. Our in vitro results clearly demonstrate that in mouse miRNAs can downregulate *F8*, much like what is observed in human HA patients. This should pave the way for studying HA and coagulation factor disorders in mice that are relevant to humans in the near future.

Preclinical animal models that have been developed for HA (rats, dogs, sheep, and genetically engineered mice and pigs) are all focused towards developing appropriate therapies for HA [7] and thus carry a non-functional *F8* gene. Only recently have we shown that miRNAs can also influence the HA disease outcomes including in patients with normal *F8* gene; to our knowledge, there are no animal models to test this hypothesis. Since mice do not develop hemophilia [11], in the currently available mouse model HA for bleeding disorder, a part or whole *F8* gene is deleted [8,20,21]. As a result, the FVIII-deficient mice have no detectable circulating factor VIII. Hence, these mouse models cannot be used to study the miRNA-based regulation of *F8*. Two transgenic mice models expressing a human FVIII variant with R593C mutation [22] and a human FIX variant with R333Q mutation [23] have been reported. However, these mice exhibit the hemophilia phenotype; i.e., the human-FVIII and human-FIX are non-functional. For models that evaluate whether microRNA mediated the control of FVIII (or FIX) expression, it is necessary that the wild-type fully functional FVIII proteins are expressed. These models can then be used to unequivocally determine whether (a) micro-RNAs can downregulate FVIII expression in vivo and (b) whether the downregulation of the *F8* gene is sufficient to elicit a hemophilia phenotype. In vivo evaluation of the effect of miRNAs on *F8* gene expression (mRNA) and subsequent FVIII production can be performed by first injecting the selected miRNAs into the tail veins similar to antibody injections as reported [24], followed by using a variety of different assays, including the tail vein transection assay as well as more recently developed models of thrombosis and hemostasis [25,26,27]. The tail transection assay measures both venous and arterial bleeding [24]. Though yet to be experimentally verified, the simplicity of this envisioned mouse model for miRNA:*F8* interactions should encourage scientists in the field to apply this approach to their research needs.

## 4. Materials and Methods

### 4.1. Cell Culture

The cell lines were obtained from ATCC. HEK293 cell lines (ATCC, CRL-11268) were maintained in Dulbecco’s Modified Essential Medium (DMEM) with 10% fetal bovine serum (FBS), 100 U/mL penicillin and 100 mg/mL streptomycin (Pen-Strep). HeLa cells (ATCC, CCL-2) were maintained in Minimum Essential Medium (MEM) with 10% FBS, and Pen-Strep and MS1 (MILE SVEN 1) (ATCC, CRL-2279) cells were grown in DMEM with 5% FBS, Pen-Strep.

### 4.2. Plasmid Constructs

Complementary DNA representing the 3’UTR of murine F8-mRNA was obtained from the liver of the C3H/HeJ mouse, amplified by RT-PCR (using primers: Mmu-*F8*-ECOR1-F: ATGCGAATTCacccccagatctgggagc and Mmu-*F8*-Not1-R: ATATGCGGCCGCaaactataagtaagccctgattaaatgc) and cloned into Gaussia luciferase reporter plasmid pEZX-MT05. pEZX-MT05 and a plasmid, pEZX-MR04-expressing precursor miRNA, along with a green fluorescent protein (eGFP) reporter gene, were both obtained from Genecopia Inc. MS2 and MS2-GST plasmids were a gift from Dr. Myriam Gorospe, NIA, NIH and described previously [13]. The murine F8 3′UTR fragment was cloned into MS2 plasmid (using primers: Mmu-*F8*-ECOR1-F: ATGCGAATTCacccccagatctgggagc and Mmu-*F8*-Not1-R: ATATGCGGCCGCaaactataagtaagccctgattaaatgc). HEK293 lines were transfected with the above vectors using the Lipofectamine 2000 transfection reagent (Invitrogen, Carlsbad, CA, USA) according to the manufacturer’s protocol.

### 4.3. Luciferase Assay

For luciferase reporter assays, HEK293 and HeLa cells were co-transfected with pEZX-MT05 plasmid encoding the muF8 3′-UTR and precursor miRNA expression plasmid to express desired miRNAs. In parallel, the cells were co-transfected with *F8* 3′-UTR pEZX-MT05-plasmid and miRNA scrambled sequence control (SC) containing pEZX-MR04. Transfection was carried out using Lipofectamine 3000 (Invitrogen, Carlsbad, CA, USA). The reporter gene activities were measured 48 and 72 h after transfection by dual-luciferase assay-system (GeneCopoeia, Rockville, MD USA). A secreted Alkaline Phosphatase (SEAP) reporter driven by a CMV promoter cloned into the same plasmid served as an internal control and was used for normalizing the transfection efficiency. The data were presented as fold-change relative to the negative (scrambled) control. MiRNA overexpression in transfected cells was quantified by qPCR [5].

### 4.4. MS2-Tagged RNA Affinity Purification (MS2-TRAP) and Identification of F8 3′UTR-Interacting miRNAs

The MS2-TRAP assay was performed as described previously [11,12,13]. Briefly, MS2-tagged *F8* 3′UTR (test) plasmid or empty MS2 plasmid (control) along with a MS2-GST plasmid expressing MS2 coat protein and glutathione transferase as a fusion protein were expressed in HEK293 cells. After 48 h, the harvested cells were lysed in NP-40 lysis buffer. Equal concentrations of clarified supernatants (2 mg/mL of lysates) were incubated with glutathione beads overnight at 4 °C to ensure binding of miRNAs to their target sequence present in the *F8* 3′UTR. Finally, the RNAs bound to the beads were extracted using Trizol reagent method, as suggested by the manufacturer’s protocol (Invitrogen, Carlsbad, CA, USA). RNA samples were analyzed in 2100 Agilent Bioanalyzer using the Small RNA kit (Agilent Technologies, Santa Clara, CA, USA) according to the manufacturer’s instructions. All analyses were performed using the Agilent 2100 expert software. RNAs from the control and test plasmid-transfected HEK293 cells were subjected to next-generation sequencing (NGS) in our in-house core facility at FDA, using an Illumina small RNA kit to identify the miRNAs associated with the *F8* 3′UTR.

The NGS data were analyzed by using miRDeep2, one of the miRNA identification tools, and then aligned this information with the miRNA database, miRBase 21. The data output was sorted based on the total number of reads for each miRNA and their fold change in the test sample relative to the control. A threshold of 1.5-fold change and read counts (read#) ≥ 40 was used to discriminate differences in the expression of miRNA. In addition, *p*-values from t-distributions were calculated for each miRNA with the assumption of standard deviation equal to 8 read counts and degrees of freedom DF = 4. A threshold of *p*-value < 0.05 was used to discriminate between significant and insignificant differences in pulled-down miRNAs.

### 4.5. Cell Transfection

Mouse MILE SVEN 1 cells (MS1 line) that endogenously express FVIII protein [14] were transfected with miRNA expression vectors using Lipophetamine 3000 (Invitrogen, Carlsbad, CA, USA). After 72 h, the cell pellet was collected and lysed either in Trizol reagent (for RNA expression studies by qPCR) or RIPA buffer (for protein expression studies by WB).

### 4.6. RT-qPCR

Total RNA from cell pellets was isolated using Trizol reagent, according to the manufacturer’s instructions. To detect the relative levels of F8-mRNA and miRNAs, quantitative real time-PCR (qPCR) was performed. For *F8*, the cDNA was generated with 1 µg total RNA by reverse transcription using SuperScript III Reverse Transcriptase (Invitrogen, Carlsbad, CA, USA) and oligo (dT) primers.

PCR products were quantitatively synthesized from cDNA samples using the TaqMan Gene Expression Master Mix and TaqMan Gene Expression Assays specific to coagulation factor VIII (Mm01215675, Applied Biosystems, Foster City, CA, USA) and GAPDH (Mm99999915, Applied Biosystems, Foster City, CA, USA). GAPDH was used as an endogenous control. The reaction conditions were used as described in gene expression master mix protocol.

For mature miRNA quantification, cDNA was synthesized from total RNA samples using specific miRNA primers provided with the TaqMan MicroRNA Assay and reagents from a TaqMan microRNA reverse transcription kit. RNU6 small nuclear RNA was used as an internal control. TaqMan miRNA assays and RNU6 snRNA were from Applied Biosystems. In the PCR step, PCR products were amplified from cDNA samples using the TaqMan MicroRNA Assay together with the TaqMan Universal PCR master mix. The reaction conditions followed TaqMan MicroRNA Assays Protocol. The fold change for each target gene relative to the control group was calculated using the ΔΔCt method.

### 4.7. Western Blot

Pellets of MS2 cells collected 48h after transfection with miRNA expression vectors were lysed by sonication in RIPA buffer with addition of PMSF (phenylmethylsulfonyl fluoride) prior to lysis. Gel electrophoresis (4–15% gradient gel) of lysates was followed by transfer to nitrocellulose membranes using Trans-Blot Turbo system (BioRad, Hercules, CA, USA). Blots were blocked with 5% skim milk in TPBS buffer (PBS buffer with Tween 20), incubated overnight with an anti-FVIII antibody (Abcam ab53703, 1:1000) followed by incubation with a secondary antibody, goat anti-Rabbit IgG, 1:10,000 (Invitrogen, Carlsbad, CA, USA) conjugated to horseradish peroxidase (HRP). Following development, blots were imaged and analyzed using an Image Station 4000MM PRO (Carestream, Rochester, NY, USA).

### 4.8. Data Analysis

Statistical analysis was performed using Microsoft Excel and Prism software. Unless otherwise indicated, statistical significance was calculated using Student’s T-test for unpaired samples, and data are presented as mean +/− Standard Error.

## Figures and Tables

**Table 1:** Micro RNAs (MiRNAs) identified in MS2-tagged RNA affinity assay. The list of 9 miRNAs detected in MS2-mu*F8* 3′UTR sample with the highest fold change compared to the control (MS2-E) with a fold change of read counts ≥ 1.5. The underlined miRNA was selected for further evaluation.

miRNA	Precursor	MS2-E	MS2-mu*F8*	Fold Change
let-7a-5p	let-7a-1	222.0	343.0	1.55
miR-93-5p	mir-93	189.0	305.0	1.61
miR-196b-5p	mir-196b	46.0	202.0	4.39
miR-196a-5p	mir-196a-1	92.0	188.0	2.04
miR-125a-5p	mir-125a	97.0	150.0	1.55
let-7f-5p	let-7f-1	55.0	147.0	2.67
miR-30e-3p	mir-30e	26.0	119.0	4.58
miR-15b-3p	mir-15b	31.0	83.0	2.68
let-7g-5p	let-7g	16.0	71.0	4.44

## Supplementary Materials

Supplementary materials can be found at https://www.mdpi.com/1422-0067/21/16/5621/s1.

## Abbreviations

DF	Degrees of freedom DF
*F8*	gene encodes coagulation factor VIII
FVIII	coagulation factor VIII
GAPDH	Glyceraldehyde 3-phosphate dehydrogenase
HEK293T	Human embryonic kidney 293 cells
HeLa	Human immortal cell line derived from cervical cancer cells
HRP	horseradish peroxidase
miRNA	micro RNA
mmu	Mus musculus (mouse)
mu*F8*	Murine *F8*
MS1	MILE SVEN 1 cells
MS2	MS2 plasmid
MS2-E	MS2 vector-empty, negative control
MS2-mu*F8*	MS2 vector fused to the 3′UTR of murine *F8*
MS2-TRAP	MS2-tagged affinity assay
NSG	Next generation sequencing
qPCR	Quantitative polymerase chain reaction
TPBS	Phosphate-buffered saline buffer with Tween 20
UTR	Untranslated region
