# Supplementary material for: A Foundational Study for Normal F8-Containing Mouse Models for the miRNA Regulation of Hemophilia A: Identification and Analysis of Mouse miRNAs that Downregulate the Murine F8 Gene
Source: Int J Mol Sci. 2020 Aug 6;21(16):5621. doi: 10.3390/ijms21165621 (PMC7460574; doi:10.3390/ijms21165621)
Supplement: Supplementary file 1 [file ijms-21-05621-s001.pdf]

International Journal of
Molecular Sciences

Supplementary Materials:

A foundational study for normal *F8*-containing mouse models for miRNA regulation of hemophilia A: Identification and analysis of mouse miRNAs that downregulate the murine *F8* gene.

Katarzyna I. Jankowska [1], Maitreyi Chattopadhyay[1,2], Zuben E. Sauna[3] and Chintamani D. Atreya[1,*]

[1] OBRR/DBCD/LCH in the Center for Biologics Evaluation and Research, US Food and Drug Administration, Silver Spring, MD 20993, USA.
[2] OTAT//DCGT/GTB in the Center for Biologics Evaluation and Research, US Food and Drug Administration, Silver Spring, MD 20993, USA.
[3] OTAT/DPPT/HB in the Center for Biologics Evaluation and Research, US Food and Drug Administration, Silver Spring, MD 20993, USA.
* Correspondence: Chintamani.atreya@fda.hhs.gov

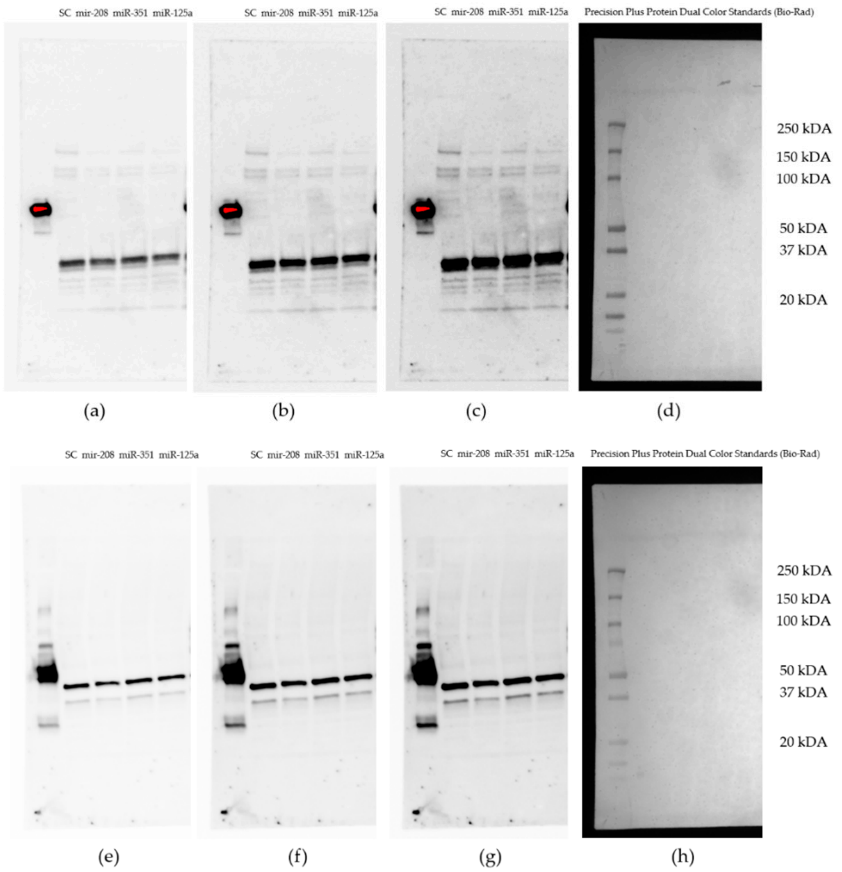

Supplemental Figure S1. Full-length blots of Figure 3c shown at three different exposures. (a-c) Relative FVIII level in MS1 cells collected 72h after transfection with miRNAs: miR-208, miR-351 and miR-125a expression vectors compared to control cells (SC) determined by Western blot form transfected samples presented at three different exposure along with molecular size marker (at d, line 1). (e-g) Loading controls: Same blots after incubation with β-actin and GAPDH antibodies at three different exposure along with molecular size marker (at h). The molecular weight of the bands were determined by Precision Plus Protein Dual Color Standards (BioRad, Hercules, CA, USA) loaded in line 1 as shown in d and h (for a-c and e-g panels, respectively).
